# HOXA genes cluster: clinical implications of the smallest deletion

**DOI:** 10.1186/s13052-015-0137-3

**Published:** 2015-04-10

**Authors:** Lidia Pezzani, Donatella Milani, Francesca Manzoni, Marco Baccarin, Rosamaria Silipigni, Silvana Guerneri, Susanna Esposito

**Affiliations:** Pediatric Highly Intensive Care Unit, Department of Pathophysiology and Transplantation, Università degli Studi di Milano, Fondazione IRCCS Ca’ Granda Ospedale Maggiore Policlinico, Via Commenda 9, Milan, 20122 Italy; Medical Genetics Laboratory, Fondazione IRCCS Ca’ Granda Ospedale Maggiore Policlinico, Milan, Italy

**Keywords:** HOXA, Speech delay, Hand-foot-genital syndrome, 7p15 deletion

## Abstract

**Background:**

HOXA genes cluster plays a fundamental role in embryologic development. Deletion of the entire cluster is known to cause a clinically recognizable syndrome with mild developmental delay, characteristic facies, small feet with unusually short and big halluces, abnormal thumbs, and urogenital malformations. The clinical manifestations may vary with different ranges of deletions of HOXA cluster and flanking regions.

**Case presentation:**

We report a girl with the smallest deletion reported to date involving the entire HOXA cluster at 7p15.2-p14.3. The patient was the third child born to a healthy and non-consanguineous Italian couple. She was born at the 34th week of gestation by caesarean section due to cholestasis of pregnancy. Her birth weight, length, and occipitofrontal circumference were 2,140 g (25-50th centile), 46 cm (50th centile), and 33 cm (75-90th centile), respectively. The Apgar scores were 8 at both the 1st and 5th minutes. The patient presented with typical mild facial anomalies, hand and feet abnormalities, urinary anomalies, and mild speech delay. Unexpectedly, the patient demonstrated complex unusual features of multiple episodes of oxyhemoglobin desaturation, laryngeal stridor and a branchial cyst. Chromosome analysis of the patient revealed an apparently normal karyotype at the 550 band level. Based on array comparative genomic hybridization, a 2.5 Mb interstitial deletion was detected at 7p15.2p14.3 (chr7: 26,333,553-28,859,312), involving the entire HOXA cluster and a small number of other genes as *SNX10, SKAP2, EVX1, HIBADH, TAX1BP1, JAZF1*, and *CREB5*.

**Conclusions:**

This report improves our understanding of the genotype-phenotype correlations of HOXA genes cluster deletions via the identification and characterization of the smallest deletion (as well as critical region) reported to date. In particular we discuss the possible implications of preterm and haploinsufficiency in the pathogenesis of the unusual findings, furthermore opening new discussion and interpretation cues.

## Background

The homeobox (HOX) genes family consists of 39 genes, which encode transcription factors playing a fundamental role in proper embryologic development. Four HOX genes clusters have been identified on different chromosomal regions in humans, the HOXA on 7p15.3, HOXB on 17q21.3, HOXC on 12q13, and HOXD on 2q31 [1]. All these four clusters are members of highly conserved gene families that are essential for the development of central nervous system (CNS), axial skeleton, limbs, gut, hematopoietic and urogenital tract, and internal and external genitalia. A number of studies stated that *HOX* genes have partially overlapping expression patterns, and consequently a considerable functional redundancy in the cited tissues [2]. Consequently, full cluster deletion might reveal any HOX cluster-specific function.

Mice lacking all *HOXA* genes functions suffer from respiratory, cardiac and hematopoietic defects which cause early postnatal death [2]. Individual mutations in some *HOXA* genes as *HOXA1*, *HOXA2*, *HOXA11*, and *HOXA13* are known to cause respectively Athabaskan brainstem dysgenesis syndrome (OMIM #601536), microtia, hearing impairment and cleft palate (OMIM #612290), radioulnar synostosis with amegakaryocytic thrombocytopenia (OMIM #605432), and hand-foot-genital syndrome (HFGS, OMIM#140000) or Guttmacher syndrome (GUTTS OMIM #176305) [1].

Only a few studies have reported the heterogeneous chromosomal aberrations of the region 7p15-p14 [3-9]. Genotype-phenotype analyses suggest that deletions involving *HOXA13* show HFGS features, which is characterized by small feet with unusually short and big toes and abnormal thumbs, and urogenital malformations. Smaller deletions of this region usually cause clinically recognizable symptoms and signs with mild developmental delay, characteristic facies, and HFGS, but larger deletions may cause more complex phenotypes, leading to difficulties of clinical diagnosis and interpretation [7-9].

Here we report a girl with a deletion of about 2.5 Mb at 7p15.2-p14.3 that contains the entire HOXA cluster. To the best of our knowledge, this is the smallest deletion involving *HOXA* genes reported to date. Unexpectedly, the patient demonstrated complex unusual features of multiple episodes of oxyhemoglobin desaturation and laryngeal stridor. The aim of this report is to compare this unusual case to previously reported HOXA cluster deletions in order to achieve a closer genotype-phenotype correlation of the only HOXA cluster deletion.

## Case presentation

The patient was the third child born to a healthy and non-consanguineous Italian couple. The pregnancy was normal, except for platelet incompatibility between the mother and fetus, treated with periodic intravenous immunoglobulin from the 29th week of gestation. The girl was born at the 34th week of gestation by caesarean section due to cholestasis of pregnancy. Her birth weight, length, and occipitofrontal circumference were 2,140 g (25-50th centile), 46 cm (50th centile), and 33 cm (75-90th centile), respectively. The Apgar scores were 8 at both the 1st and 5th minutes. She received nasal continuous positive airways pressure (NCPAP) ventilation for the first six days due to frequent oxyhemoglobin desaturation, which recurred in the first months especially during feeding, but gradually improved. Bilateral frontal pseudocysts were detected by transfrontal ultrasonography and confirmed by brain magnetic resonance imaging (MRI). She suffered from bradycardia and sporadic supraventricular extrasystoles, without visible anomalies based on echocardiography examination. Renal ultrasonography detected bilateral bifid pelvis with left vesicoureteral reflux (VUR) of grade III. At night she suffered from laryngeal stridor that gradually improved and disappeared. A fibroscopy of the larynx was performed and excluded any structural defects of the larynx. She also suffered from gastroesophageal reflux disease (GERD) that was improved by Omeprazole and Domperidone, but recurred at three years of age.

**Figure 1:**
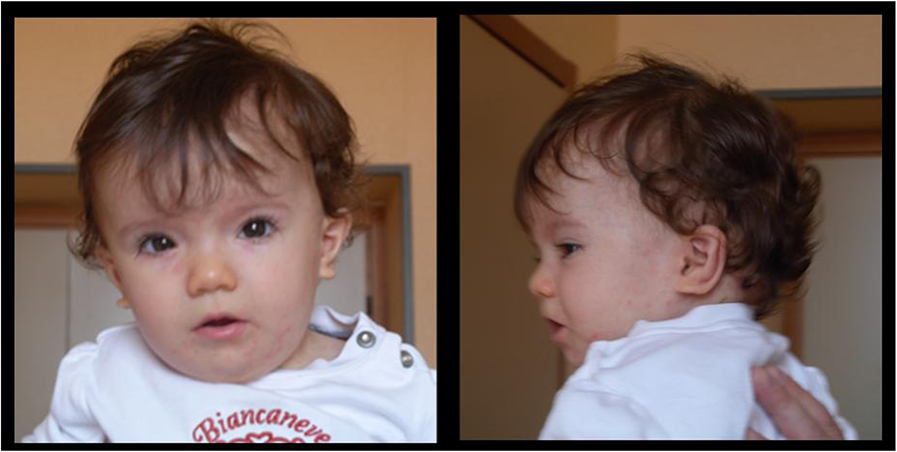
**Characteristics of the facial gestalt of the patient: frontal bossing, mild hypertelorism, short palpebral fissures, wide nasal bridge, long philtrum, thin lips, micrognathia, prominent anti-helix, uplift of ear lobules, and broad and short neck.**

**Figure 2:**
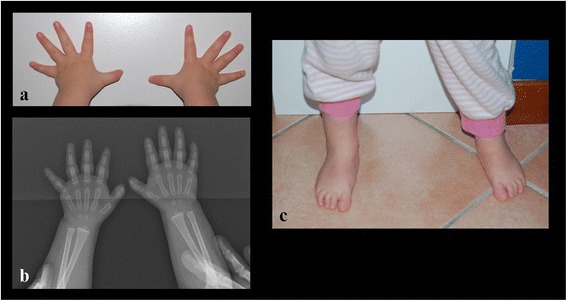
**Hands showing tapering fingers and mild bilateral hypoplasia of the distal phalanges of the first and second finger (part a), confirmed by X-ray (part b); small feet with brachydactyly (part c).**

A poor growth was evident, while the psychomotor development was normal with the exception of a mild speech delay. The physical examination at 1 year of age showed short stature (<3rd centile, −2.5 SD) and relative macrocephaly (75-90th centile). She exhibited facial dysmorphisms such as frontal bossing, mild hypertelorism, short palpebral fissures, wide nasal bridge, long philtrum, thin lips, micrognathia, uplift of ear lobules, prominent anti-helix, and broad and short neck (Figure 1). She also showed small feet with brachydactyly. Ophthalmological or audiological involvement and genital anomalies were not detected, and complete blood count and chemistry panel were all within the normal range. Hand X-ray showed mild bilateral hypoplasia of the distal phalanges, especially of the first and second fingers (Figure 2). In addition, the parents noted a small cervical mass, which was identified as a branchial cyst by ultrasound examination. A clinical re-evaluation at 3 years of age confirmed the reported findings and the presence of a normal IQ with only an isolated mild speech delay, evaluated with Griffiths Mental Development Scales.

**Figure 3:**
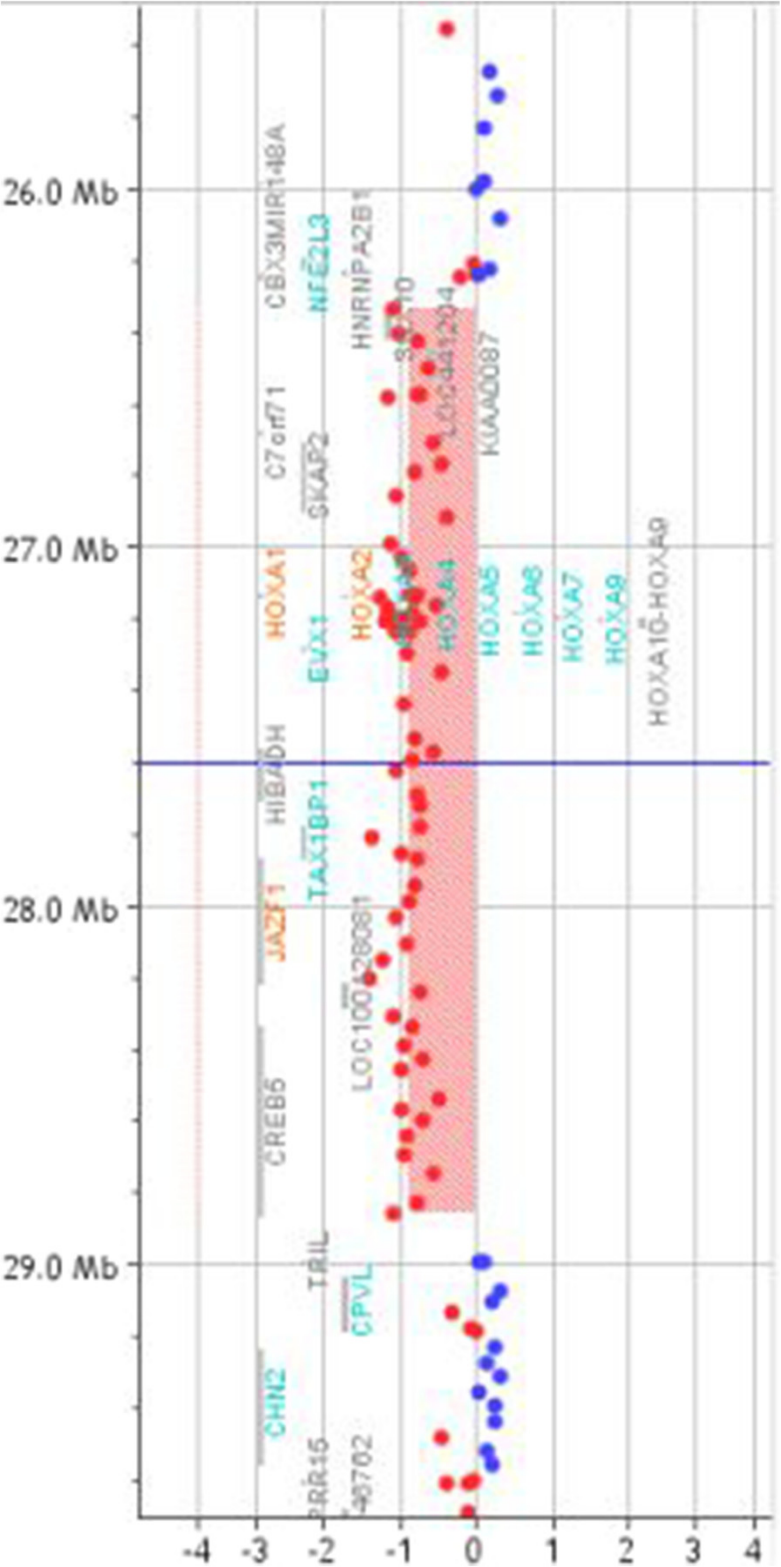
**Array CGH results of the patient: a 2.5 Mb deletion at 7p15.2p14.3 involving the HOXA cluster genes.** The breakpoints are according to the 37 build (March 2009) of the Human Genome Reference Consortium (GRch37/hg19).

Conventional karyotyping of peripheral blood lymphocytes was carried out according to the standard laboratory procedure. Chromosome analysis was performed at 550 banding level, according to the ISCN [10] and the European General Guidelines and Quality Assurance for Cytogenetics [11] and revealed an apparently normal karyotype. Based on a-CGH, a 2.5 Mb interstitial deletion was detected at 7p15.2p14.3 (chr7: 26,333,553-28,859,312), involving the entire HOXA cluster and a small number of other genes as *SNX10, SKAP2, EVX1, HIBADH, TAX1BP1, JAZF1*, and *CREB5* (Figure 3). This deletion was not identified in her parents, indicating the *de novo* origin of the alteration. Submicroscopic genomic alterations were detected by array comparative genomic hybridization (a-CGH) on genomic DNA from peripheral blood lymphocytes, using Agilent SurePrint G3 human 8x60K kit according to the manufacture’s instruction. Data were analysed using CytoGenomics 2.0.6.0 (Agilent Technologies, Cernusco sul Naviglio, Milan, Italy). Aberrations were considered if at least three adjacent probes were involved. Copy number variations were not reported if they are listed in the Database of Genomic Variants (http://projects.tcag.ca/variation/). This study was approved by the Ethics Committee of the Fondazione IRCCS Ca’ Granda Ospedale Maggiore Policlinico, Milan, Italy, and written informed consent was obtained from patient’s parents.

## Conclusions

**Figure 4:**
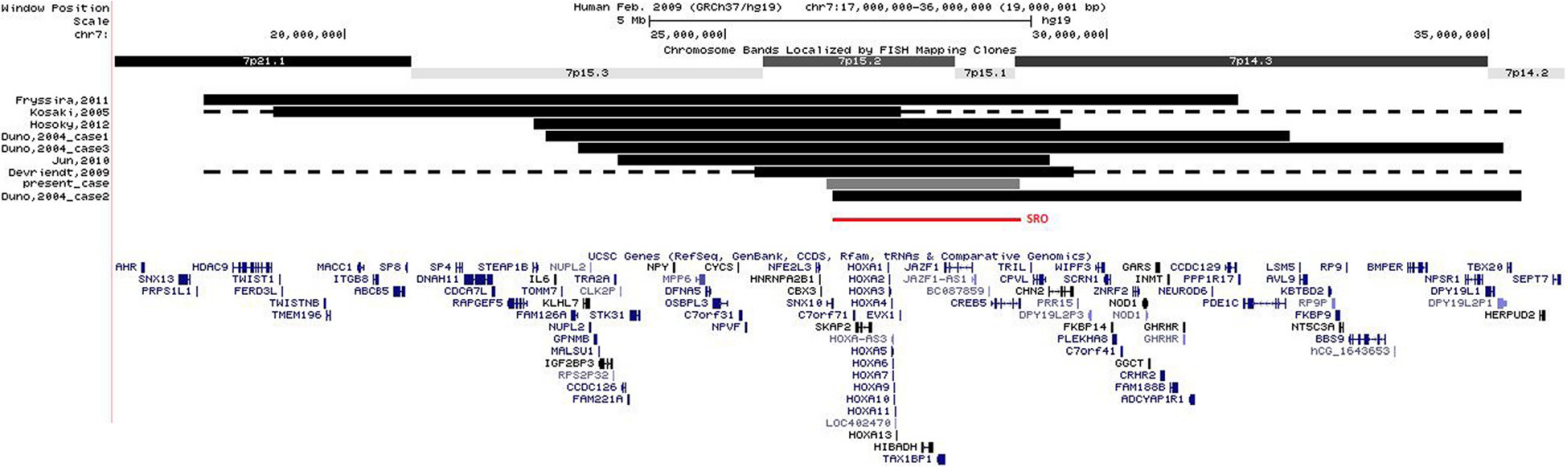
**Overlapping deletions of the present and of the previous cases.** The dotted line means that the exact breakpoints of these patients are unknown (Kasaki [7] and Devriendt [5]). The new smallest region of overlap (26,408,229-28,859,312) is defined by the distal breakpoint of the deletion of Dunø’s patient 2 n and the proximal breakpoint of the aberration of the present case. The breakpoints are according to the 37 build (March 2009) of the Human Genome Reference Consortium (GRch37/hg19).

All previously reported deletions involving *HOXA13* show HFGS features, namely bilateral and symmetrical limb malformations with incompletely penetrant urogenital defects, and some additional findings in comparison to mutation of the single gene, such as mild dysmorphic facies, developmental delay, feeding difficulties in the first months of life, and mild intellectual disability. These observations are based on clinical findings of seven patients with deletions of about 8.8 ± 3.2 Mb (the deletion described by Kosaki [7], was detected by FISH, so the breakpoints are not available, but it was visible at G-banding chromosome analysis) encompassing the entire HOXA cluster (Figure 4; Table 1) [3-7]. Here we report a girl with a 2.5 Mb deletion at 7p15.2-p14.3, which is the smallest deletion among all previous reports, involving the entire HOXA cluster and seven additional genes.

**Table 1:** **Genetic and clinical features of the patients included in this study and previous reports**

	**Patient 1**	**Patient 2**	**Patient 3**	**Patient 4**	**Patient 5**	**Patient 6**	**Patient 7**	**Patient 8**
	**Present case 3y, female**	**Devriendt et al., 1999** ** [** 5 **]** **21y, male**	**Dunø et al., 2004** **[** 6 **]** **5y, female**	**Dunø et al., 2004** **[** 6 **]** **4y, male**	**Kosaki et al., 2005** **[** 7 **]** **1y, male**	**Jun et al., 2011** **[** 3 **]** **4y, male**	**Hosoki et al., 2012** **[** 4 **]** **13y, male**	**Fryssira et al., 2011** **[** 8 **]** **2y, male**
**Chromosomal deletion**	7p15.2p14.3	7p15.3p14.2	7p15.3p14.3	7p15.2p14.2	7p15.2p21	7p15.3p15.1	7p15.3p15.1	7p21.1p14.3
**Size (Mb)**	2.5	12	9,8	9	NA	5,6	6,9	13
**Inheritance**	De novo	De novo	De novo	De novo	De novo	De novo	De novo	De novo
**Neonatal feeding problem**	Yes, due to GERD	Yes, due to velopharingeal insufficiency	NA	NA	NA	Yes, due to dysphagia	Yes	Yes, due to GERD
**Developmental delay**	Yes	No	Yes	Yes	Yes	Yes	Yes	Yes
**Intellectual disability**	No	Borderline IQ	Yes	Yes	Yes	Yes	No	Yes
**Speech delay**	Yes	No	Yes	Yes	Yes	Yes	Yes	Yes
**Ear anomalies**	Uplift of ear lobules, hyperplastic anti-helix	Low-set, malformed	NA	Short, slightly deformed	Prominent ear crus	Low-set, posterior angulated, mild EAM stenosis	Low-set, posterior rotated	Low-set, Helix hypoplasia, hyperplastic anti-helix and anti-tragus, prominent intertragic notch
**Palpebral fissures**	Short	Upslanted	Upslanted	Dowslanted	NA	Upslanted	No	Short, epicanthic folds, ptosis
**Other facial anomalies**	Frontal bossing, mild hypertelorism, wide nasal bridge, long philtrum, thin lips, micrognathia, short neck	Retrognathia, upturned nostrils, large mouth	Flat nasal bridge, broad nose, anteverted nostrils, broad lips	Broad neck	Depressed supraorbital ridge on the left, maxillary hypoplasia	Flat nasal bridge, frontal bossing	Bifrontal narrowing	Asymmetry, hypertelorism, low nasal bridge, anteverted nostrils, long and smooth philtrum, short neck
**Small hands and feet**	Small feet	NA	Yes	Yes	No	Yes	Yes	Yes
**Clinodactyly**	No	Yes	Yes	Yes	No	No	Yes	Yes
**Other fingers anomalies**	Hypoplasia of distal phalanges of thumbs and 2nd fingers	Pointed distal phalanx	Short fingers	Short fingers	Hypoplasia of 5th fingers	Limited extension	No	Digital webbing and abnormal hand creases
**Great toe anomalies**	No	Shortened, laterally deviated	Short and broad	NA	No	Hypoplasia	Curved and broad	Relatively long, broad and medially deviated
**Other toes anomalies**	Short toes	Triangular distal phalanx	NA	Short toes, pes planus	No	No	Short	Short
**Genital anomalies**	No	Cryptorchidism, ventral-bowed penis	NA	No	Rectoperineal fistula	Hypospadia	No	Hypospadia, left cryptorchidism and scrotum hypoplasia
**Urinary anomalies**	Bifid pelvis with left VUR	No	No	No	No	No	No	Moderate renal insufficiency
**Velopharyn-geal anomalies**	No	Yes	No	No	No	No	No	Yes
**Heart malformations**	No, only bradycardia and sporadic supraventricular extrasystoles	Patent ductus Botalli	No	No	Patent ductus arteriosus	Patent ductus arteriosus, partial pulmonary venous return	No	Open foramen ovale, aortic insufficiency
**Other features**	Desaturation episodes, GERD, stridor	No	No	Hypermetropia	Anal atresia, cranio-synostosis	Accessory nipples	No	Craniosynostosis, desaturation episodes (abnormal sleep EEG)

EAM: external acoustic meatus; GERD: gastroesophageal reflux; IQ: intelligence quotient; y: year-old; NA: not available; VUR: vesicoureteral reflux; EEG: electroencephalogram.

Hosoki [4] tried to compare the previous reports and found a small region of overlap (SRO; chr7: 26,374,754-29,360,960) involving nine genes plus the HOXA cluster, and defined it as the critical region. This region almost completely overlaps the alteration of our patient, but exceeds 535 kb containing 2 more genes (*CVPL* and *CHN2*). So, we can now redefine and reduce the former critical region.

The girl in the present study showed a number of typical manifestations of *HOXA13* deletion, such as speech delay, short stature (below the 3rd centile), VUR, small feet with brachydactyly, bilateral mild hypoplasia of distal phalanges of some fingers of the hands, and some mildly dysmorphic facial features that were highly similar to the case 2 reported by Dunø [6].

In the present study, the psychomotor development of the patient was normal, except for a mild speech delay. It is notable that different degrees of intellectual impairments have been reported to date, that goes from borderline [4,5] or even normal IQ, to severe disability [3].

Interestingly, our patient exhibited a number of additional features, such as bradycardia and sporadic supraventricular extrasystoles in absence of heart malformations, GERD, oxyhemoglobin desaturation during feeding, and laryngeal stridor. These manifestations, together with frontal pseudocysts, may be related to preterm delivery. Respiratory distress caused by oxyhemoglobin desaturation [12], especially during feeding, as well as GERD, bradycardia [13], and frontal pseudocysts, are indeed well documented in preterms [14]. However, interestingly, one of the *HOXA* genes, *HOXA5,* is known to be essential for organogenesis and function of respiratory tracts [15]. Moreover in homozygous newborn mutant mice, abnormal morphogenesis of respiratory tracts and tracheal occlusion have been observed, which led to respiratory distress immediately after birth [15].

Loss of *HOXA3* function in mouse results in larynx anomalies that are similar to the phenotypes caused by *HOXA5* mutations*.* Homozygous defects of *HOXA3* actually seem to cause lethal cardiovascular dysfunctions, loss and malformations of throat cartilages and jaw bones, disorganization of the throat musculature, absent thymus and parathyroids, and thyroid hypoplasia [16,17], while heterozygous defects are associated to ectopic thymus and/or parathyroids [18] so haploinsufficiency of this gene has been suggested [5] as the cause of these anomalies. The presence of a cervical branchial cyst in our patient, never pointed out in the previous reported cases, is intriguing too, given the cited role of *HOXA3* in the differentiation of neck tissues.

The previously reported cases of velopharyngeal insufficiency and dysphagia [3,5], and the manifestations of our patient may be related to the haploinsufficiency of *HOXA3* and/or *HOXA5* as already stated by Devriendt [5]; alternatively in our case, they may be explained by the additive effects of haploinsufficiency and mild prematurity.

Concerning the other genes involved in our deletion, only *JAZF1* and *SNX10* were designated as morbid genes, according to the OMIM database (http://www.ncbi.nlm.nih.gov/omim/). *JAZF1* is known as participant in the fusion gene resulting from recurrent t(7;17) (p15;q21) translocation in endometrial stromal sarcoma [19], while homozygous mutations in *SNX10* cause autosomal recessive Osteopetrosis [20].

It is notable that in 2004 Lehoczky et al. [21] demonstrated that *EVX1*, *HIBADH*, *TAX1BP*, *JAZF1* and *CREB5* show embryonic distal limb and genital bud expression, but at this time it is not known whether they have a role in their development.

In conclusion, this report improves our understanding of the genotype-phenotype correlations of HOXA genes cluster deletions via the identification and characterization of the smallest deletion (as well as critical region) reported to date, furthermore opening new discussion and interpretation cues on the unusual findings outlined.

## Consent

Written informed consent was obtained from the patient’s parents for publication of this Case report and any accompanying images. A copy of the written consent is available for review by the Editor of this journal.

## Abbreviations

a-CGH: array comparative genomic hybridization
CNS: Central nervous system
GERD: Gastroesophageal reflux disease
GUTTS: Guttmacher syndrome
HFGS: Hand-foot-genital syndrome
HOX: Homeobox
MRI: Magnetic resonance imaging
NCPAP: Nasal continuous positive airways pressure
SRO: Small region of overlap
VUR: Vesicoureteral reflux
